# Predicting stunting in Rwanda using artificial neural networks: a demographic health survey 2020 analysis

**DOI:** 10.12688/f1000research.141458.1

**Published:** 2024-02-20

**Authors:** Similien Ndagijimana, Ignace Kabano, Emmanuel Masabo, Jean Marie Ntaganda

**Affiliations:** 1 African Centre of Excellence in Data Science, Kigali, Kigali, Rwanda; 2 College of Business and Economics, University of Rwanda, Kigali, Kigali, Rwanda; 3 College of science and Technology, University f Rwanda, Kigali, Kigali, Rwanda

**Keywords:** Feature importance, Artificial Neural Networks, Stunting, Children, Rwanda.

## Abstract

**Background:**

Stunting is a serious public health concern in Rwanda, affecting around 33.3% of children under the age of five in 2020. Several examples of research have employed machine learning algorithms to predict stunting in Rwanda; however, no study used artificial neural networks (ANNs), despite their strong capacity to predict stunting. The purpose of this study was to predict stunting in Rwanda using ANNs and the most recent Demographic and Health Survey (DHS) data from 2020.

**Methods:**

We used a multilayer perceptron (MLP) architecture to train and test the ANN model on a subset of the DHS dataset. The input variables for the model included child, parental and socio-demographic’s characteristics. The output variable was a binary indicator of stunting status (stunted *vs.* not stunted).

**Results:**

An overall accuracy of 72.0% on the test set was observed, with an area under the receiver operating characteristic curve (AUC-ROC) of 0.84, indicating the model’s good performance. Several factors appear as important contributors to the probability of stunting among the negative value aspects. First and foremost, the mother’s height is important, as a lower height suggests an increased risk of stunting in children. Positive value characteristics, on the other hand, emphasie elements that reduce the likelihood of stunting. The timing of the initiation of breastfeeding stands out as a crucial factor, showing that early breastfeeding initiation has been linked with a decreased risk of stunting.

**Conclusions:**

Our findings suggest that ANNs can be a useful tool for predicting stunting in Rwanda and identifying the most important associated factors for stunting. These insights can inform targeted interventions to reduce the burden of stunting in Rwanda and other low- and middle-income countries.

## Introduction

Stunting remains a significant public health issue worldwide, particularly in low-and middle-income countries. According to the latest estimates by the World Health Organisation (WHO), in 2020, around 149.2 million children under the age of five years (about 22% of all children in this age group) were affected by stunting globally, with the highest burden in low and middle-income countries. ^1^ The COVID-19 pandemic has exacerbated the situation, since disruptions in food systems and health services are likely leading to an increase in stunting rates. ^2^ Therefore, stunting is still a serious public health problem across the world, particularly in Africa. ^2^ In 2020, about 58 million children under the age of five were stunted in Africa, accounting for nearly 40% of all stunted children worldwide. The prevalence of stunting in these EAC countries highlights the importance of focused interventions to address the underlying causes of stunting, which include poverty, poor nutrition, and a lack of access to healthcare and sanitary services. ^3^

Artificial Neural Networks (ANNs) are a type of machine learning (ML) technique that has gained prominence in recent years due to its ability to learn and generalise from data, making them ideal for predictive modelling applications. ^4^ ANNs are a kind of deep learning, which is a technique that consists of training models with numerous layers of connected nodes to replicate human brain function. ^4^ ANNs stand out in significance when contrasted with other ML algorithms for a multitude of compelling reasons. In many circumstances, ANNs can simulate complicated, non-linear interactions between input and output variables, allowing for accurate predictions. ^5^ Linear models, such as linear regression, have limitations in capturing nonlinear correlations, but more complex models, such as decision trees or random forests, may overfit the data or be computationally costly, however, ANNs perform better. ^6^ ANNs are usually resistant to noisy or incomplete data, making them useful in real-world situations where data is frequently poor. ^6^ ANNs can automatically extract significant features from data, removing the need for manual feature engineering. This can save time and effort while modelling, especially with high-dimensional data. ^7^ ANNs are suited for big data applications because they can be scaled to accommodate massive datasets, hence, it is critical to apply them to Rwanda Demographic Health Survey (RDHS). ^7^ The RDHS is nationally representative research that collects data on a number of health indicators, including stunting, through house interviews. ^8^

They can also be parallelised over many processors, increasing computing efficiency. ^9^ ANNs may be used for transfer learning, which involves fine-tuning a pre-trained model for a new task with little data. This is especially important when data is scarce or expensive to collect. ^9^ Using an ANN to predict stunting offers various advantages, including increased accuracy and the capacity to identify significant predictors of stunting. ANNs can analyse vast volumes of data and detect patterns that typical statistical approaches may miss, allowing for more accurate stunting predictions. Furthermore, ANNs can identify major predictors of stunting, such as poverty, low maternal education, and a lack of access to sanitary facilities, allowing for focused interventions to address the core causes of stunting. ^10^ A few studies have been conducted in Rwanda using other machine learning technics like logistic regression, Supportive Vector Machine (SVM), Naive Bayes Random Forest (RF), XGBoost gradient model. ^11^

However, based on the existing knowledge there has been few researches in Rwanda that attempted to utilise ML to predict stunting like the study conducted by Similien *et al.* ^11^ ANNs have proven to be highly effective in predicting illnesses. However, Similien’s publication did not delve into the utilisation of ANNs for this purpose, despite their demonstrated effectiveness in prediction. Recognising this gap, a supplementary study was essential to explore the application of ANNs in predicting stunting in children, using data from the 2020 RDHS. Given the crucial importance of addressing the root causes of stunting for effective treatments and policymaking, the researcher chose to conduct this study on the application of ANNs in the specific context of stunting in Rwanda, using the same dataset as the aforementioned publication. ^11^ The remaining party of this study is organised as follow: Methods, Results, Discussion, Conclusion and recommendation.

## Methods

DHS is a large-scale household survey program that is carried out in low- and middle-income nations. DHS surveys are meant to collect high-quality data on health, demographic, and nutrition indicators to help policymakers and program administrators make better decisions. The surveys are normally conducted every five years and give information on a variety of areas including fertility, mother and child health, family planning, HIV/AIDS, nutrition, and gender-based violence. ^12^ The secondary data from the 2019-2020 RDHS were analysed in this study. The RDHS is a five-year quantitative, cross-sectional study-based national survey. The RDHS used a two-phase stratified sampling approach. In the first step, 500 clusters were chosen from a pool of 112 urban enumeration areas and 388 rural enumeration areas. In the second stage, homes were systematically sampled, involving the selection of a random subsample of 26 households within each cluster, resulting in a total of 13,000 surveyed households. This subsample specifically included 3,814 children under the age of five, from whom height and weight measurements were collected. ^13^

### Quantitative variables

**Explanatory variables**

The explanatory factors for stunting that were associated with the characteristics of mothers, households, and children are summarised below ( Table 1). The selection of variables from the DHS was guided by UNICEF conceptual framework children nutrition and tailored to the specific context of Rwanda. ^14^

**Table 1.** Explanatory factors are assessed and documented for use in the analysis.

Variables	Description	Categories
**Variables related to a child’s characteristics**		
**Baby’s age**	Age of the child in months	0: <6, 1: 6-11, 2: 12-23, 3: 24-35, 4: 36-47, 5: 48-59
**Sex**	Sex of the child	0: female, 1: male
**Size of a child**	Size of the child at birth	0: large, 1: average, 2: small
**Birthweight**	The weight of the child at birth	0: ≥2.5 kg, 1: <2.5 kg
**Breastfeeding start**	Time when the child starts breastfeeding	0: within the first hour, 1: 1-24 hours, 1-2: 30 days
**Presence of diarrhoea**	The child had diarrhoea in the last 2 weeks	0: No, 1: Yes
**Variables related to the child’s mother**		
**Maternal age**	Age of the mother	0: less than 18 years, 1: Between 19-35 years, 2: greater than 35 years
**Maternal education**	Education level of the mother	0: no education, 1: primary, 2: secondary or higher
**Maternal anaemia**	Anaemia status the of mother	0: not anaemic, 1: anaemic
**Marital status**	Mother’s marital status	0: single, 1: married, 2: separated
**Maternal height**	Respondent’s height in centimetres	0: <160 cm, 1: ≥160 cm
**Antenatal**	Number of antenatal visits during pregnancy	0: no antenatal care, 1: 1-4 antenatal care visits, 2: more than 5 antenatal care visits
**Variables related to households**		
**Residence**	Type of place of residence of the child	0: rural, 1: urban
**Source of drinking water**	Source of drinking water in the household	0: unimproved, 1: improved
**Toilet facilities**	Type of toilet facilities in the household	0: unimproved, 1: improved
**Place of delivery**	Distribution of live births by place of delivery	0: other, 1: delivery at home, 2: delivery at health facility
**Province**	Region	0: Kigali, 1: south, 2: east, 3: west, 4: north
**Reading**	Frequency of reading newspapers or magasines	0: ever, 1: reading
**Altitude**	Cluster altitude in meters	0: ≤2000 m, 1: >2000 m

DHS, Demographic and Health Surveys; WHO, World Health Organisation.

**Outcome variable**

The outcome variable in this study was stunting status, which was classified according to WHO criteria. The nutritional status of children was separated into two categories based on height for age z-scores, as follows: stunted if standard deviation SD<−2 was less than the median, and not stunted otherwise. ^13^

### Data preprocessing

Data preprocessing is the activity of preparing (cleaning and arranging) raw data so that it is understandable and useable for analysis. It consists of various stages, including data cleansing, data integration, data transformation, and data reduction. ^15^ In this study, data cleaning was characterised as the procedure for addressing missing or incomplete data within the dataset. The missing values were addressed through imputation using the K Nearest Neighbours (KNN) imputer, which, when contrasted with the Euclidean distance, might result in a reduction of data similarity. ^16^ Data transformation here used to describe the process of altering the format or structure of data to make it suitable for analysis, ^15^ where the researcher encoded categorical data with the map function before converting it to dummy (0 and 1) values with pandas (pd). Obtain dummies that treated variable categories individually, then use the Minimax scaler to normalise the numerical data, which ranges all data values between 0 and 1, code was generated in Python using the popular ML library scikit-learn. Moreover, the Synthetic Minority Over-Sampling Technique (SMOTE) was employed to tackle the class imbalance within the target variable. This technique involves oversampling the minority class by generating synthetic instances along the line segments that connect any or all of the k nearest neighbours within the minority class. ^17^ The software used during the data preprocessing and analysis was the python Google Collab. ^18^

### Training dataset

A dataset is divided into three subsets: a training set, a validation set, and a test set. The training set is used to train the model, the validation set is used to fine-tune the model’s hyperparameters and avoid overfitting, and the test set is used to assess the model’s final performance on new data. Each subset’s size is determined by the amount of the dataset and the model’s complexity. The dataset, consisting of 3814 observations, was divided into 80% for training (3051 instances) and 20% for testing and validation (763 instances).

### Artificial neural networks model

The ANN was built using 23 inputs to predict stunting in Rwanda. After initialising the neural network, the model employed neurons as features in the input layer and two in the hidden layer. Two hidden layers were used in the ANN, a common choice for optimising performance, and they were tested individually to determine the ideal configuration for achieving the desired results in this proposed model. Because the goal of this study is to identify stunted newborns using training data, the rectifier activation function in the hidden layers and the sigmoid activation function in the output layer are used to set a range (0, 1) of a linear function in ANN. ^19^ 80% and 20% of the data have been used as training and testing data respectively for a model that runs 100 epochs. Each epoch is seen as having one forward and one backward propagation. Finally, the most effective stochastic gradient descent optimiser parameter “Adam” is employed. The batch size is set to 32, which implies there are 10 occurrences in each epoch at any given moment. The loss (binary- crossentropy) function is used to classify the losses. With ANN, the best outcome is offered after computing the loss. ^20^^,^^21^

### Model training and evaluation

To train the ANN model on the training set using the hyperparameters chosen. During the training phase, the model’s capacity to recognise complicated patterns in the data is constantly refined. We rigorously monitor two critical parameters throughout this training process: loss and accuracy. Loss measures how much our model’s predictions differ from the real values, whereas accuracy measures how frequently the model’s predictions match the actual outcomes. Early stopping is a strategy in which the model’s performance is evaluated on a distinct dataset called the validation set on a frequent basis during training. This collection is unique from the training data and is used to assess the model’s generalisation capabilities. Model evaluation, on the other hand, is used to assess the performance of the trained ANN model on the test set.

The primary metrics used for assessment were accuracy, precision, recall, and the area under the receiver operating characteristic curve (AUC-ROC). This metric quantifies the overall correctness of the model’s predictions by measuring the ratio of correctly predicted stunting to the total children. Precision assesses the accuracy of positive predictions of stunting made by the model. It is calculated as the ratio of true positive predictions to the sum of true positives and false positives. Recall, also known as sensitivity or true positive rate, evaluates the model’s ability to capture all relevant instances. It is calculated as the ratio of true positives to the sum of true positives and false negatives. The AUC-ROC provides a comprehensive evaluation of the model’s ability to discriminate between stunted and no stunted children. A higher AUC-ROC value indicates superior discrimination performance. The analyses were conducted using the TensorFlow and scikit-learn libraries in Python. ^22^

### Features importance

**Figure 1.**
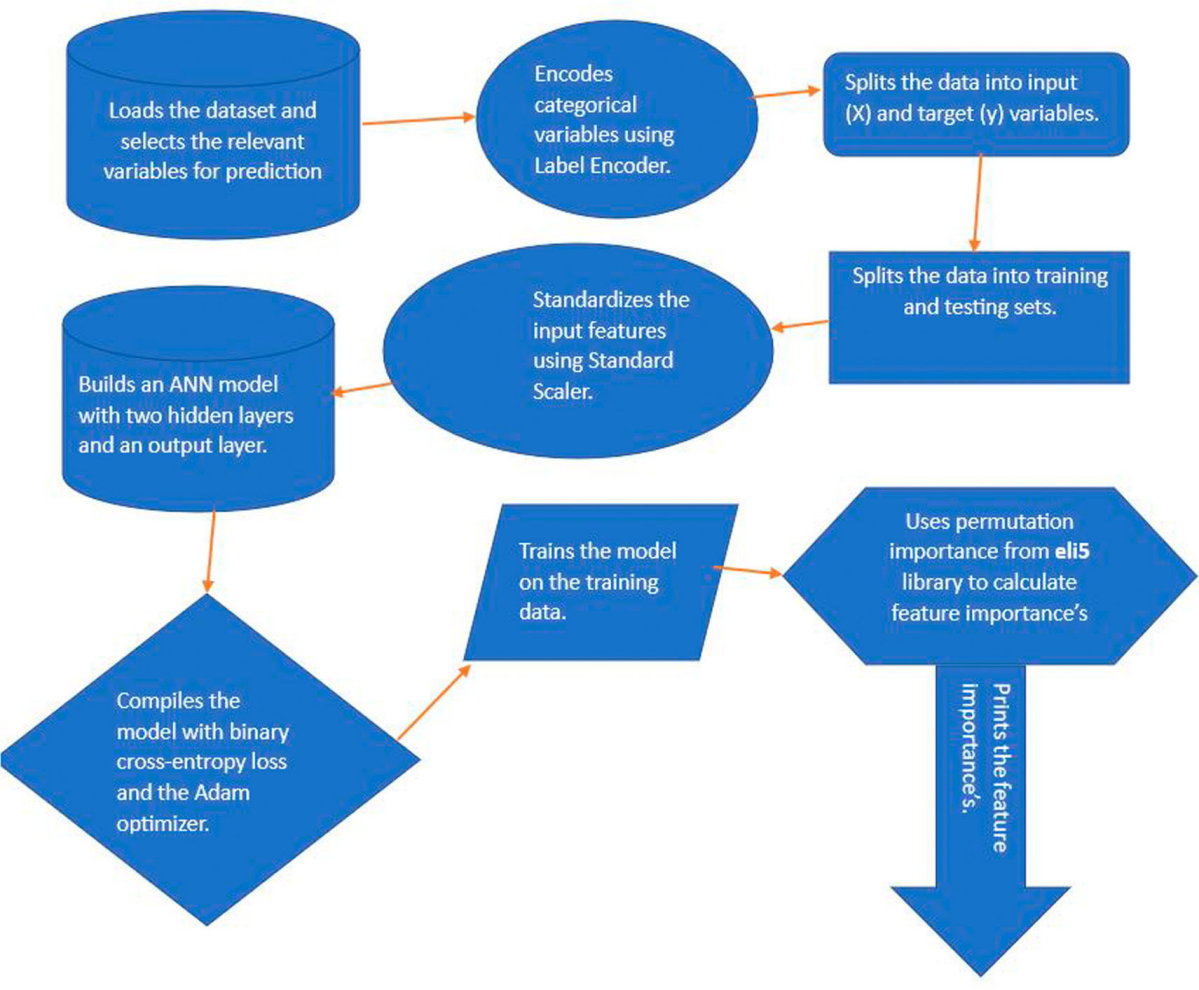
The 10 steps to calculate and print list of feature importance using ANN.

Here are 10 steps that we used for feature selection as seen in Figure 1: Step 1 includes importing the dataset and picking the necessary columns for prediction. In this case, the dataset has 23 input features and the ‘stunting’ variable as the target variable. Step 2 the function LabelEncoder is used to encode categorical variables. LabelEncoder is a scikitlearn (RRID:SCR_002577) utility class that encodes category characteristics as numeric values; Step 3 entails separating the data into input (X) and target (Y) variables. The input characteristics are contained in the X variable, while the target variable is contained in the Y variable; Step 4 entails dividing the data into 80% for training (3051 instances) and 20% for testing and validation (763 instances). The ANN model is trained using the training set, and its performance is evaluated using the testing set; Step 5 using StandardScaler, standardise the input characteristics. The scikit-learn library’s StandardScaler utility class standardises features by eliminating the mean and scaling to unit variance. Step 6: Build an ANN model with two hidden layers and one output layer. The number of neurons in the input layer is equal to the 23 of input features; Step 7 specifically, in our stunting prediction model, we opted for the Adam optimiser and binary cross-entropy loss. The choice of the binary cross-entropy loss function is crucial for binary classification tasks, such as distinguishing between instances of stunting and non-stunting. This loss function quantifies the difference between the predicted and actual outcomes, providing a measure of how well the model is performing in terms of classification accuracy; in Step 8, the model is trained using a dataset comprising a 3814 number of observations. In this case, the training data used for model training involves a determined amount of information. The model is exposed to this dataset over a series of iterations known as epochs. In each epoch, the model refines its weights and biases based on the training data to improve its predictive capabilities. The choice of 100 epochs ensures that the model undergoes a sufficient number of iterations to converge and achieve optimal performance. Additionally, a batch size of 32 is employed, signifying that the model processes 32 instances of data in each epoch before updating its parameters. This batch-wise training approach helps in optimising computational efficiency and contribute to the model’s generalisation ability.

Step 9 to determine feature importance, the eli5 library’s permutation importance is used. Permutation importance was the model-independent strategy used after training an ANN to predict stunting. It measures the decline in model performance when each feature is randomly shuffled across instances to determine the relevance of particular characteristics. The decline in performance, as measured by measures like as accuracy or AUC-ROC, reflects the significance of a feature. Permutation is performed over numerous samples, and the procedure is iterated to ensure consistency. Features that cause a significant decline in performance are thought to be critical for the ANN’s prediction accuracy in stunting situations. This strategy assists in the identification and prioritisation of critical elements that influence the model’s predictions. For scikit-learn and Keras models, the eli5 module implements permutation importance. ^21^ Step 10 includes printing the features’ importance. The features’ importance is printed in descending order of significance, along with their appropriate weights.

## Results

**Figure 2.**
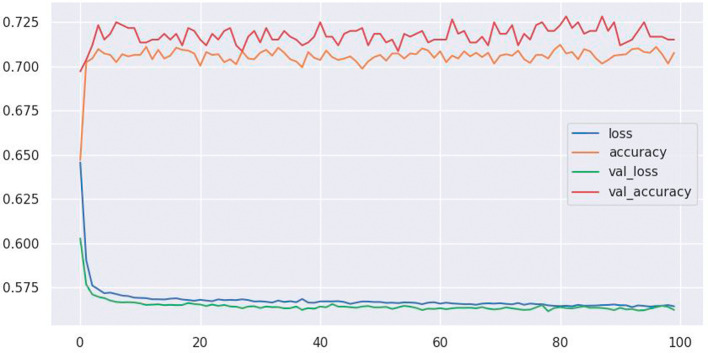
Model accuracy.

This section shows the results from ANN model, Figure 2 shows an accuracy of 72%.

**Figure 3.**
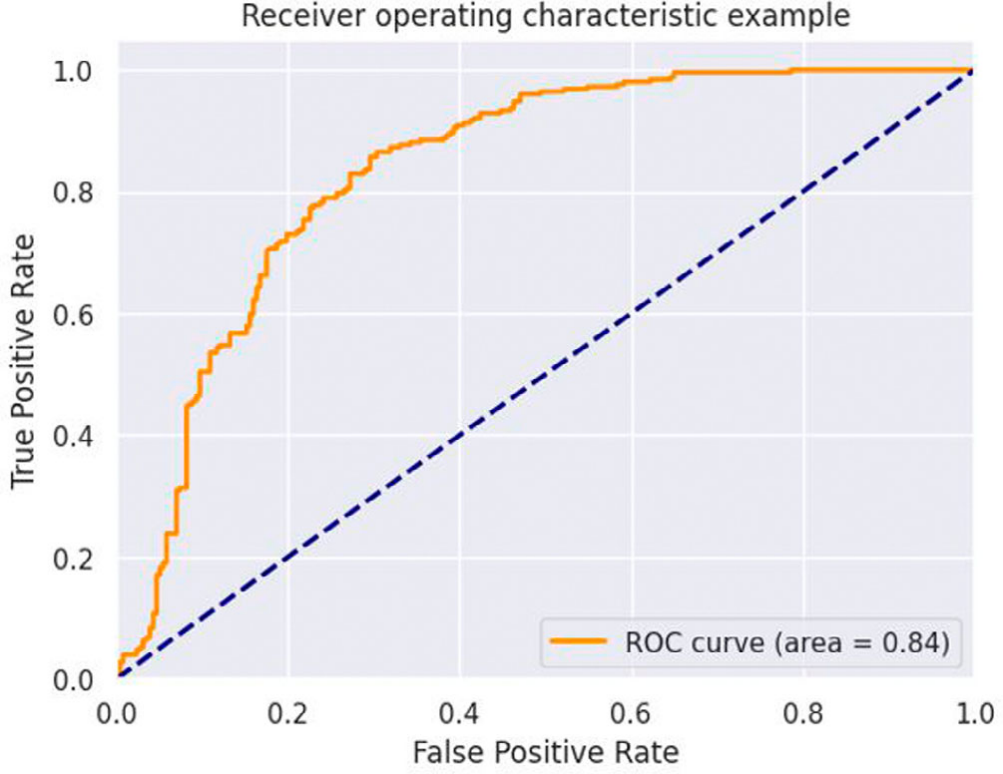
ROC curves of Artificial Neuron Network.

The ANN model built in this study showed good results in predicting stunting in Rwandan children. The model attained a 72% accuracy and a ROC of 0.84, indicating that it is a very useful tool for detecting children at risk of stunting as shown in Figure 3.

**Figure 4.**
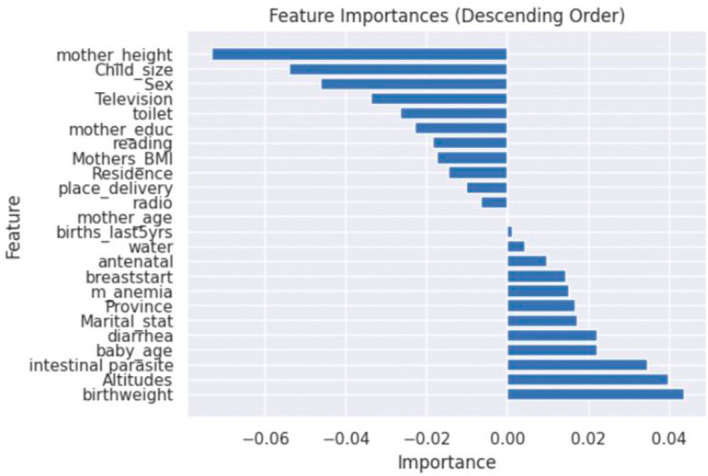
Feature importance using ANN model.

The feature importance analysis of an ANN model for predicting stunting in Rwanda was examined in this article. The model reveals the important elements related to stunting, giving light to both negative and positive value characteristics. The features of importance were computed and displayed using the ANN model as seen in Figure 4. Identifying these positive and negative value characteristics is pivotal for comprehending the intricate dynamics of stunting and devising targeted interventions to mitigate its prevalence.

### Negative value aspects

Figure 4 shows the realm of stunting prediction, certain factors exhibit a negative correlation with the likelihood of a child experiencing stunting. These include the mother’s height, child’s size at birth, gender of the child, mother’s education, place of residence, reading newspapers, and the mother’s age. These aspects, when present, tend to indicate a reduced risk of stunting.

### Positive value characteristics

There are characteristics associated with a positive correlation, suggesting an increased risk of stunting in children. These positive value features encompass the initiation of breastfeeding, the presence of mother’s anaemia, marital status, province of residence, occurrences of diarrhoea, altitude, birthweight, and the age of the baby as shown in Figure 4.

## Discussion

The ANN model’s results show considerable promise. The model displays its capacity to correctly categorise cases of stunting and non-stunting within the dataset with a significantly high degree of precision, with an accuracy of 72%. This demonstrates the model’s ability to detect critical patterns and variables associated with stunting in Rwandan children as also shown by Uddin et al. ^23^ This paper also show that ANN is very powerful compared to other models. ^24^ Furthermore, the model’s ROC score of 0.84 demonstrates its excellent discriminative capabilities. A higher ROC score indicates better distinction between the stunted and non-stunted child. The algorithm excels at reliably rating stunted children above non-stunted children, as indicated by an ROC score of 0.84, which is crucial in identifying individuals at high risk. These findings highlight the ANN model’s potential as a useful tool for predicting and mitigating stunting in Rwanda. ^25^ This study reveals that it is crucial to note that the ROC curve and AUC-ROC should be assessed in conjunction with other assessment measures like accuracy, precision, and recall to provide a thorough picture of the model’s performance and applicability for practical application in stunting prediction, however, this study used only the ROC. ^25^

Early identification of children at risk of stunting allows treatments to be targeted to those who need them the most, decreasing the burden of stunting on both the individual and society as a whole. ^26^ The use of ANN in stunting prediction offers the potential to improve early detection and intervention tactics. Policymakers and healthcare professionals may prioritise targeted treatments and deploy resources more efficiently if the primary factors leading to stunting are identified. ^27^ The study’s findings suggest that focusing on initiatives to enhance maternal nutrition, promote breastfeeding practices, and improve access to high-quality healthcare services could be of paramount importance in addressing the identified risk factors highlighted in the research as shown in Figure 4. Breastfeeding start appears as an important element, showing that early breastfeeding starting is connected with a decreased chance of stunting. These findings agree with the study conducted with Saberi-Karimian et al. ^27^ Understanding the factors that contribute to childhood stunting gives vital information to policymakers, healthcare professionals, and communities. ^28^

The same as the finding of this study suggested that efforts should be directed at improving maternal nutrition and health, supporting exclusive breastfeeding practices, and guaranteeing access to healthcare services that successfully manage maternal anaemia as well as the prevention and treatment of diarrhoea disorders, the mother’s height is important, as a lower height suggests an increased risk of stunting in children. ^29^ Additionally, customised treatments should be developed for certain provinces with a greater rate of stunting. The awareness among mothers and caregivers about the importance of child proper nutrition, breastfeeding practices, hygiene, and the significance of regular healthcare visits should be increased. ^30^ The research recommended that regular health checkups, growth monitoring, and testing to detect potential growth and developmental delays should be improved. Early measures, including nutritional supplements, caregiver counselling, and relevant healthcare interventions, can then be administered. ^31^ The gender of the child is also associated with the outcome of the stunting of the child as seen in Figure 4. The mothers giving birth to boys should pay close attention to the nutrition of their babies, as different studies revealed boys are more stunted than girls. ^32^

Being at higher altitudes is associated with a high risk of stunting in children in Rwanda as revealed by the study. ^11^ It is crucial to emphasise that altitude is only one of several factors that contribute to stunting, and its influence varies depending on other contextual factors such as economic status, healthcare facilities, and dietary choices. ^33^ To alleviate stunting in high-altitude locations, a comprehensive approach is required, which includes increasing access to healthcare, nutrition, sanitation, and education, as well as addressing the underlying socioeconomic determinants of health, ^34^ this study also confirm the findings shown in the different studies. Finally, encourage collaboration among government agencies, healthcare providers, non-governmental organisations, and community-based organisations in order to execute comprehensive and multi-sectoral stunting elimination strategies. This partnership can ensure a comprehensive strategy to address the recognised stunting feature importance by combining the expertise and resources provided by different stakeholders. ^34^

However, while the model worked well in this study, it is possible that it may not generalise well to other populations or circumstances. Further study is needed to test the model’s generalisability and to uncover other factors that may improve its performance. Another limitation pertained to the discussion phase, primarily due to the scarcity of existing ANN literature focused on stunting prediction. Consequently, it was challenging to draw meaningful comparisons between this research and prior studies. The contribution of this research is the use of ANN analysis in stunting prediction in Rwanda results in improved identification of significant characteristics, real-time monitoring, targeted interventions, and useful policy decision-making assistance. These contributions increase our understanding of stunting, guide targeted interventions, and may eventually contribute to lowering stunting rates and enhancing children’s well-being in Rwanda.

## Conclusions

The feature significance analysis of the ANN model in predicting stunting in Rwanda demonstrates the intricate interaction of numerous factors in affecting child growth and development. Positive value features stress the relevance of breastfeeding habits, mother health, and socioeconomic variables, whereas negative value features emphasise the importance of maternal qualities, education, and environmental factors. Rwanda may adopt targeted interventions and policies to minimise stunting prevalence and promote healthy growth and development for its children by addressing these major causes. In conclusion, the ANN model developed in this study provides a promising approach to predicting stunting in Rwanda. With further validation and refinement, it has the potential to significantly contribute to efforts aimed at reducing the stunting prevalence and improving child health outcomes in the country.

## Data Availability

In this study, we analysed datasets that are publicly available in the DHS program repository. https://www.dhsprogram.com/data/dataset_admin/login_main.cfm. The request was address to DHS program after providing the purpose of the use of data then after we obtained authorisation from the DHS administration to use them. Data access is granted to individuals who create an account and submit a request. The DHS team reviews the request and, upon approval, provides access to the requested data.

## Software availability

Source code available from: https://github.com/Similien1/ANN_stunting/blob/main/Copy_of_Artificial_Neuron_Network.ipynb

Archived source code: https://doi.org/10.5281/zenodo.8424559. ^18^

Declaration: This manuscript shares the same data as the paper published https://jpmph.org/journal/view.php?doi=10.3961/jpmph.22.388 however the methodology used and result obtained are different.
