# Supplementary material for: Predicting stunting status among under-5 children in Rwanda using neural network model: Evidence from 2020 Rwanda demographic and health survey
Source: F1000Res. 2025 Jan 20;13:128. Originally published 2024 Feb 20. [Version 2] doi: 10.12688/f1000research.141458.2 (PMC11840296; doi:10.12688/f1000research.141458.2)
Supplement: Peer review history

# Reviewer report: Reviewer response for version 2

**DOI:** 10.5256/f1000research.176526.r360888

**Published:** 2025-02-19

**Authors:** Mediana Aryuni

**Affiliations:** 1 Bina Nusantara University, Jakarta, Indonesia

The author has already revised the input from the reviewer.

Is the work clearly and accurately presented and does it cite the current literature?

Partly

If applicable, is the statistical analysis and its interpretation appropriate?

Yes

Are all the source data underlying the results available to ensure full reproducibility?

Partly

Is the study design appropriate and is the work technically sound?

Partly

Are the conclusions drawn adequately supported by the results?

Partly

Are sufficient details of methods and analysis provided to allow replication by others?

Partly

Reviewer Expertise:

NA

I confirm that I have read this submission and believe that I have an appropriate level of expertise to confirm that it is of an acceptable scientific standard.

# Reviewer report: Reviewer response for version 2

**DOI:** 10.5256/f1000research.176526.r360889

**Published:** 2025-02-11

**Authors:** Mashfiqul Huq Chowdhury

**Affiliations:** 1 Mawlana Bhashani Science and Technology University, Santosh, Bangladesh

**Comments**

 I have reviewed the revised paper along with the authors’ responses to previous comments.

 While I am generally satisfied with their responses, I believe several areas still need improvement. My detailed comments are outlined below.

 1. Check for grammatical errors.

 2. Provide the full forms of EAC and ANN before introducing their abbreviations for the

 fist time in the article.

 3. Artificial Neural Networks (ANN) are the cornerstone of deep learning. The author describes ANN as a machine learning method. Since ANN and multilayer perceptron

 refer to the same concept, I recommend revising the sentences related to ANN in the Abstract and Introduction sections to eliminate redundancy.

 4. The last sentence of the Introduction needs correction. It starts with ‘The remaining party of this study is organized as follows....

 5. The author should maintain consistency in using ‘k-nearest neighbors’ and ‘Minimax scaler’ throughout the article.

 6. The authors have used secondary data from the 2019–2020 RDHS; however, the title mentions only the 2020 RDHS.

 7. I assume the outcome variable is not properly defied. Stunting status is typically defied when the HAZ score is less than -2 SD. Please verify the definition of the HAZ score and revise the sentence accordingly.

 8. The author mentions using Python Google Collab for data analysis. The correct phrasing should b e ’Python’ instead.

 9. The authors divide the dataset into three subsets: a training set, a validation set, and a test set. However, in the last sentence, they state that the dataset, consisting of 3,814 observations, was divided into 80% for training and 20% for testing and validation (763 observations). They need to specify the exact sizes of the validation and test datasets.

 10. The authors need to specify the sizes of the two hidden layers used in the ANN.

 11. The authors should specify which deep learning package they used (e.g., PyTorch or TensorFlow) along with the version number of each packages and software. Additionally, including a reference for the Adam optimizer would be beneficial.

 12. There is an inconsistency in the description of the neural network architecture. On page 6, the authors state, ‘After initializing the neural network, the model employed neurons as features in the input layer and two in the hidden layer.’ However, on page 10, they mention, ‘In this study, a multi-layer perceptron (MLP) uses one hidden layer.’ To avoid confusion, I recommend clarifying the number of hidden layers used in the model and ensuring consistency throughout the manuscript.

 13. On page 6, I suggest changing the heading ’Artificial Neural Networks Model’ to ’Experimental Setup’ to better reflect the content of the section.

 14. On page 6, the following sentence may not b e generally true: ‘Two hidden layers were used in the ANN, a common choice for optimizing performance.’ I recommend revising this sentence for better coherence and accuracy, as the optimal number of hidden layers depends on the specific problem and dataset.

 15. The authors state, ‘Step 10 includes printing the features’ importance.’ However, it is unclear whether this conclusion is based on the training data or test data. Please

 clarify this. I suggest presenting feature importance using the test data for a more reliable evaluation.

 16. I suggest including the confusion matrix to better demonstrate the test accuracy and provide a more comprehensive evaluation of the model’s performance.

 17. In Figure 4, the predictor variable labels on the Y-axis should b e written in their full form. For example, if ’mother height’ is used, I recommend changing it to ‘mother’s height’ for better readability and clarity.

 18. The following sentence seems more appropriate for the Introduction rather than the Discussion section:

 ’The potential use of ANN is not only in stunting prediction but also in other healthcare services, such as an optimum ANN-based breast cancer diagnosis. ANNs have been used for various data classification and pattern recognition tasks in medical image processing and have shown promise as a classification tool for breast cancer. The use

 of neural networks in image and signal processing grew substantially in the early 1980s.’ I recommend moving this content to the Introduction to provide better context for the

 study.

 19. The Conclusion section should be more concise while effectively conveying key findings and future research directions. I recommend streamlining the content to focus on the main contributions of the study and potential areas for further exploration.

 .

Is the work clearly and accurately presented and does it cite the current literature?

Yes

If applicable, is the statistical analysis and its interpretation appropriate?

Partly

Are all the source data underlying the results available to ensure full reproducibility?

Yes

Is the study design appropriate and is the work technically sound?

Yes

Are the conclusions drawn adequately supported by the results?

Yes

Are sufficient details of methods and analysis provided to allow replication by others?

Partly

Reviewer Expertise:

Statistics and Data Science

I confirm that I have read this submission and believe that I have an appropriate level of expertise to confirm that it is of an acceptable scientific standard, however I have significant reservations, as outlined above.

# Reviewer report: Reviewer response for version 1

**DOI:** 10.5256/f1000research.154905.r274788

**Published:** 2024-06-12

**Authors:** Mediana Aryuni

**Affiliations:** 1 Bina Nusantara University, Jakarta, Indonesia

The paper needs some justifications for these concerns:

1.  The reason why they only applied 1 Machine learning algorithm (ANN), while their previous research (11) applied 6 algorithms?
2. The reason why they chose kNN for data imputation and SMOTE for data balancing
3. Reconsider differentiating validation and testing data.
4. Feature importance is done before or after learning model development
5. Figure 1 is not systematic and clear
6. It will be interesting if they also compare the accuracy with and without SMOTE
7. They must explain why ANN doesn't perform better than Random forest, SVM, extreme gradient boosting, and gradient boosting in the discussion.

Is the work clearly and accurately presented and does it cite the current literature?

Partly

If applicable, is the statistical analysis and its interpretation appropriate?

Yes

Are all the source data underlying the results available to ensure full reproducibility?

Partly

Is the study design appropriate and is the work technically sound?

Partly

Are the conclusions drawn adequately supported by the results?

Partly

Are sufficient details of methods and analysis provided to allow replication by others?

Partly

Reviewer Expertise:

NA

I confirm that I have read this submission and believe that I have an appropriate level of expertise to confirm that it is of an acceptable scientific standard, however I have significant reservations, as outlined above.

# Response

**Published:** 2024-12-31

**Authors:** Similien NDAGIJIMANA

**Affiliations:** Primary health care, University of Rwanda, Butare, Southern Province, Rwanda

1. The reason was explained in the background of this paper “Given the crucial importance of addressing the root causes of stunting for effective treatments and policymaking, the researcher chose to conduct this study on the application of ANNs in the specific context of stunting in Rwanda, using the same dataset as the aforementioned publication”
2. Choosing kNN for data imputation ensures that missing values are filled in a manner that maintains the natural relationships within the data, while SMOTE for data balancing addresses class imbalance, enhancing the model's ability to learn from and predict both stunted and non-stunted cases effectively. These preprocessing steps are crucial for building a robust and reliable ANN model for stunting prediction in Rwanda.
3. The validation and testing were differentiated in the methodology section 
4. The feature importance was done after model development
5. I am sorry I did not get what did you mean by this question is it to refine the diagram? I keep it as it was however the title has been changed 
6. I did however I get the same before balancing and after balancing 

While ANNs have shown substantial effectiveness in various predictive tasks, including illness prediction, their performance may not always surpass that of other algorithms like Random Forest, SVM, XGBoost, and Gradient Boosting in specific contexts, such as stunting prediction. The reasons include the higher complexity and risk of overfitting, significant data requirements, sensitivity to data quality, need for extensive feature engineering, and the inherent strengths of other algorithms in handling structured data, computational efficiency, and ease of hyperparameter tuning. Thus, the choice of algorithm should be guided by the specific characteristics of the dataset and the problem at hand.

# Reviewer report: Reviewer response for version 1

**DOI:** 10.5256/f1000research.154905.r274789

**Published:** 2024-05-22

**Authors:** Mashfiqul Huq Chowdhury

**Affiliations:** 1 Mawlana Bhashani Science and Technology University, Santosh, Bangladesh

This study employs a deep learning model to predict stunting status among children under the age of five in Rwanda. The findings are intriguing. However, several improvements are necessary. The comments are detailed below:

**Title** can be modified to: Predicting stunting status among under-5 children in Rwanda using neural network model: Evidence from 2020 Rwanda demographic and health survey

 In the Abstract, the results section needs improvement. The authors should rewrite the following sentence: Several factors appear as important contributors to the probability of stunting among the negative value aspects.

**Introduction section:**

 a) Discuss the consequences of stunting and the overall situation of stunting in Rwanda among under-5 children.

 b) In the second paragraph, the author critically evaluates the linear regression and decision tree models. It is recommended that the author also discuss the logistic regression model.

 c) This sentence is not relevant to me: "They can also be parallelised over many processors, increasing computing efficiency.9 ANNs may be used for transfer learning, which involves fine-tuning a pre-trained model for a new task with little data. This is especially important when data is scarce or expensive to collect." 

 d) Spelling mistake: Supportive Vector Machine

 e) Give a reference to this sentence: ANNs have proven to be highly effective in predicting illnesses.

 f) The authors need to provide a more detailed description of the motivation behind this study.

 g) The literature review is very limited. I suggest adding more references to existing studies and relevant literature.

 h) This sentence is not clear: "Given the crucial importance of addressing the root causes of stunting for effective treatments and policymaking, the researcher chose to conduct this study on the application of ANNs in the specific context of stunting in Rwanda, using the same dataset as the aforementioned publication.

**Methods section:**

 a) This section can be split into the following sub-sections: Data Source and Sampling Design, Study Population, and Variables (Explanatory and Outcome Variables). The authors need to discuss the data file used from the DHS website, including information on any missing variables and whether they were discarded or handled in some other way.

 b) In Table 1, the following changes are recommended: replace "Baby's age" with "Child's age," "Antenatal" with "Antenatal care visits," and "Reading" with "Media access." For variables such as source of drinking water, toilet facilities, and place of delivery, define the categories clearly in the variables sub-section. Specify the definitions for unimproved and improved statuses earlier in the text. Also, clarify which places are considered health facilities. Instead of using mother's height, the authors may consider the mother's BMI variable.

 c) The variables "size of a child" and "birthweight" are essentially the same. Should both variables be included?

 d) The authors need to clarify why the altitude variable is crucial for this analysis.

 e) Authors can create a new subsection titled "Experimental Setup" to systematically outline the experimental procedures. This section should be written in a clear and organized manner to ensure ease of understanding for readers.

 f) In the section discussing the Artificial Neural Network (ANN) model, it's important to specify the size of the hidden layer and provide justification for this choice, possibly citing relevant references. Additionally, authors should explain how they tuned the hyperparameters of the model. Including a reference for the Adam optimizer would also be beneficial. To improve clarity and prevent redundancy, it's recommended not to repeat the discussion of Scikit-learn, TensorFlow, and Python many times.

 g) The authors should clarify the context in which they use both standardization methods, Minimax scaler and Standard scaler, in their paper. It's important to explain why each method is chosen and how they are applied to the data. This clarification will help readers understand the rationale behind using different scaling techniques and their impact on the results.

 i) Use observations instead of instances.

 j) Figure 1 title needs to be improved.

**Results section:**

 a) In Figure 2, the x-axis and y-axis titles should be clearly labeled. The legends also need to be improved to indicate "Training Loss," "Test Loss," "Training Accuracy," and "Test Accuracy." Additionally, the main title of the figure should be rewritten for clarity.

 b) Write a general comment based on Figures 2, 3, 4. Write in 2-3 paragraphs without sub-sections.

 c) If feasible, conduct a comparison with a machine learning model like logistic regression. This comparative analysis will elucidate which type of model (ML or DL) is best suited for this type of dataset.

 d) This sentence is not clear to me: "This study reveals that it is crucial to note that the ROC curve and AUC-ROC should be assessed in conjunction with other assessment measures like accuracy, precision, and recall to provide a thorough picture of the model’s performance and applicability for practical application in stunting prediction, however, this study used only the ROC".

**Discussion section:**

 Authors can emphasize identifying similarities or consistency with other existing works.

Is the work clearly and accurately presented and does it cite the current literature?

Yes

If applicable, is the statistical analysis and its interpretation appropriate?

Partly

Are all the source data underlying the results available to ensure full reproducibility?

Yes

Is the study design appropriate and is the work technically sound?

Yes

Are the conclusions drawn adequately supported by the results?

Yes

Are sufficient details of methods and analysis provided to allow replication by others?

Partly

Reviewer Expertise:

Statistics and Data Science

I confirm that I have read this submission and believe that I have an appropriate level of expertise to confirm that it is of an acceptable scientific standard, however I have significant reservations, as outlined above.

# Response

**Published:** 2024-12-31

**Authors:** Similien NDAGIJIMANA

**Affiliations:** Primary health care, University of Rwanda, Butare, Southern Province, Rwanda

**For title**

 The title is modified as suggested

**For abstract**

 I added some results, however, there is a limited number of words

 The sentence has been rephrased like this: Factors appear to contribute to stunting among the negative value aspects. 

**Introduction section:**

1. The discussion was added
2. As in the paper I published before and due to the limitation of the article I did not discuss this model here.
3. The sentence is removed
4. Supportive is changed into supporting
5. The reference is given
6. More details were given
7. The references are added
8. I paraphrased this sentence

**Methods section:**

1. The subsections have been added
2. The variables were changed
3. The variables "size of a child" and "birthweight " are not almost the same since one measures the height while the other measures the weight.
4. From the analysis made before we find that altitude contributes to stunting being at high altitude is an associated risk factor for stunting, even in this analysis, ANN shows altitude as the associated factor to stunting.
5. The section was not added due to the limited number of words however were explained in the methods
6. The comment was accepted and adjusted as suggested.
7. The clarification is given
8. The comments are considered the **observation replaced the instances **
9. The title is improved  

**Results section:**

 a) The title and legends have been added

 b) I did it as suggested

 c) the comparison made in the previous study

 d) The sentence is paraphrased,

 “This study highlights the importance of evaluating the ROC curve and AUC-ROC alongside other metrics such as accuracy, precision, and recall to gain a comprehensive understanding of the model's performance and its practical applicability in predicting stunting. However, it is important to note that this study only used the ROC for assessment.

**Discussion section:**

 The emphases on similarities or consistency with other existing works were added

# Reviewer report: Reviewer response for version 1

**DOI:** 10.5256/f1000research.154905.r274786

**Published:** 2024-05-17

**Authors:** Billy Ogwel

**Affiliations:** 1 Kenya Medical Research Institute- Center for Global Health Research (KEMRI-CGHR), Kisumu, Kenya

This manuscript addresses a significant public health challenge and provides valuable insights. To further enhance its impact, the authors may consider the following suggestions:

**Abstract:**

 -Add detail on the modelling approach in the methods section.

 - Report 95% CI around the estimates

 -Be very explicit on the targeted interventions in the conclusion of the abstract.

**Introduction**

 -More recent estimates of stunting are available 148.1 million as at 2022 ( https://www.who.int/data/gho/data/themes/topics/joint-child-malnutrition-estimates-unicef-who-wb)

 -The authors can add successful implementations of ANNs in the healthcare domain in the paragraph where they talk about the strengths of ANNs.

 -The authors need to add the justification of their study why it is important to use ANNs to the same dataset that was used by authors in reference 11 in the same setting using SVM, NB, RF, LR, and XGBoost algorithms . Do they aim to achieve a higher predictive accuracy?

**Methods**

 The authors say the data was partitioned into a training set, a validation set, and a test set. Yet talk only of a 80%:20% split. They could clarify this.

**Results:**

 -The authors could refer to the TRIPOD checklist on reporting of diagnostic or prognostic prediction studies (Collins GS ,et.al., 2015 [Ref1]) . Specifically, they could : "Describe the flow of participants through the study, including the number of participants with and without the outcome and, if applicable, a summary of the follow-up time. A diagram may be helpful."

 -They also need to report performance measures (with CIs) for the prediction model according to this guideline.

**Discussion:**

 The authors could improve the discussion by comparing their results to those of other studies that have used DHS to predict stunting and particularly reference 11 that was done in the same setting using the same data. Did their ANN model improve performance as hypothesized in the background?

 the authors could also discuss the potential clinical use of the model, highlighting where and when it can be used in the healthcare system

Is the work clearly and accurately presented and does it cite the current literature?

Partly

If applicable, is the statistical analysis and its interpretation appropriate?

Yes

Are all the source data underlying the results available to ensure full reproducibility?

Yes

Is the study design appropriate and is the work technically sound?

Yes

Are the conclusions drawn adequately supported by the results?

Yes

Are sufficient details of methods and analysis provided to allow replication by others?

Yes

Reviewer Expertise:

Enteric Research, health informatics

I confirm that I have read this submission and believe that I have an appropriate level of expertise to confirm that it is of an acceptable scientific standard, however I have significant reservations, as outlined above.

## References

[1] : Transparent reporting of a multivariable prediction model for individual prognosis or diagnosis (TRIPOD): the TRIPOD Statement. *BMC Med* .2015;13: 10.1186/s12916-014-0241-z 1 10.1186/s12916-014-0241-z 25563062 PMC4284921

# Response

**Published:** 2024-12-31

**Authors:** Similien NDAGIJIMANA

**Affiliations:** Primary health care, University of Rwanda, Butare, Southern Province, Rwanda

**For the abstract**

 The modelling approach has been added 

 - The percentage mentioned in the abstract is derived from the figure; therefore, the 95% confidence intervals have not been provided.

 - The target interventions have been explained in detail in the abstract, however, due to the limited number of words more have been given in the conclusion 

**For introduction**

 - The reference and the estimated number of stunted children were added in the introduction section

 -The successful applications of ANN have been added

 - The reason was explained in the background of this paper “Given the crucial importance of addressing the root causes of stunting for effective treatments and policymaking, the researcher chose to conduct this study on the application of ANNs in the specific context of stunting in Rwanda, using the same dataset as the aforementioned publication”

**For Methodology**

 -This explanation clarifies that the validation set is a subset of the training data, not the testing data, which helps to refine and tune the model before the final evaluation of the test set. Hence testing and validation share 20%

**For results**

 -The proportion of Stunted children was given in the results section

**For Discussion**

 -The incorporation of Artificial Neural Networks (ANN) within various healthcare applications has been integrated into the discourse; nevertheless, due to the constraints of the paper's length, I have included some additional insights.
